# Empathetic Leadership in Corporate Communication: Cultivating Positive Dynamics and Enhancing Employee Well-Being

**DOI:** 10.3390/bs16030412

**Published:** 2026-03-11

**Authors:** Karen Robayo-Sanchez, Michael A. Cacciatore, Juan Meng

**Affiliations:** Grady College of Journalism and Mass Communication, University of Georgia, Athens, GA 30602, USA; karen.robayosanchez@uga.edu (K.R.-S.); mcacciat@uga.edu (M.A.C.)

**Keywords:** public relations, empathetic leadership, work engagement, organizational commitment, employee engagement

## Abstract

This study aims to examine the impact of empathetic leadership in corporate communication, focusing on its role in enhancing employee well-being and fostering a positive workplace culture. It explores how empathetic communication contributes to trust, engagement, and long-term organizational success. Based on an international survey conducted among communication professionals in Canada and the United States (n = 1055), our analyses revealed significant gender disparities in the perception of empathy among senior communication leaders, with male professionals reporting higher perceived empathy compared to female professionals. Additionally, hierarchical position influenced perceptions, with higher-ranking employees reporting stronger empathic leadership. Perceptions of increased empathic communication over the past year were notably higher among men, older employees, and those with more experience. Empathetic leadership demonstrated a strong positive correlation with employee engagement and organizational commitment but did not significantly impact burnout. Findings from this study contribute to a broader understanding of how leadership empathy varies across professional environments and demographic groups, underscoring the complex dynamics of gender and organizational structure in shaping workplace experiences. Findings in our study contribute to both the advancement of leadership theory and the improvement of corporate communication practice.

## 1. Introduction

In the fast-paced and ever-evolving landscape of the public relations (PR) industry, effective leadership is paramount to navigating complexities and driving success. Central to this leadership paradigm is the concept of empathetic communication, wherein leaders demonstrate understanding, compassion, and emotional intelligence in their interactions with stakeholders. Empathy, as a fundamental component of effective communication, is a relational practice through which leaders cognitively and affectively attune to others’ emotions and perspectives and adapt their communicative responses to foster inclusion, trust, and mutual understanding among stakeholders. Empathetic leadership in corporate communication practice goes beyond traditional notions of authority and command, emphasizing the importance of building genuine connections, fostering trust, and cultivating a supportive organizational culture. Empathy functions as a critical leadership mechanism by allowing leaders to recognize, interpret, and respond to others’ needs and emotions within complex organizational relationships. As the PR industry continues to evolve in response to shifting societal, technological, and cultural dynamics, the role of empathetic leadership becomes increasingly pertinent in driving organizational success and maintaining competitive advantage. Understanding and harnessing empathy not only enhances leaders’ ability to connect with internal and external stakeholders but also influences organizational outcomes such as employee engagement, burnout, and organizational commitment (Kock et al., 2019). However, the dynamics of empathetic leadership in the PR industry are multifaceted, shaped by factors including cultural nuances, organizational excellence, gender dynamics, and the evolving landscape of remote and flexible work arrangements.

Drawing on recent research insights, this study aims to explore the nuanced interplay between empathetic leadership and its implications on corporate communication within the PR industry. To date, the bulk of the work on empathetic leadership has been situated in general business contexts. This work explores empathetic leadership in a specific and understudied sector, that of communication. While all specialty fields carry unique demands that warrant empathetic leadership, communication specialists are often tasked with producing content under intense time pressures. Such sensitivity to time likely makes empathetic leadership particularly important and worthy of study. Further, this work explores the topic in multiple countries to determine how consistent any of the observed patterns in the data are across cultures. Some specific research objectives include examining variations in perceived empathy among senior communication leaders by several critical demographic factors, such as country, organizational excellence, gender, type of organization, and hierarchical level within the organization. Additionally, this research aims to investigate the impact of empathetic communication on employee engagement, burnout, intention to change roles, and affective organizational commitment, shedding light on the implications for organizational practice and future research endeavors.

## 2. Literature Review

### 2.1. Overview of the Development and Evolvement of Empathetic Leadership

Various theories emphasize the significance of empathy in effective leadership. According to Avolio and Bass (1995), empathy allows leaders to demonstrate personalized consideration towards their followers. Rubin et al. (2005) suggest that empathy enables leaders to identify and understand emotions in others, while Walumbwa et al. (2008) argue that it fosters self-awareness and awareness of the context.

Empathy is a keystone in effective crisis communication. It’s the ability to understand and resonate with others’ feelings, pivotal for building trust, reassurance, and connection during turbulent times. As Abrams (2020) notes, leaders who offer transparent communication and emotional grounding help reduce collective anxiety and maintain organizational confidence in times of crisis. Additionally, studies by Dolamore et al. (2021) emphasize how empathetic communication can mitigate the psychological impact of crises, thus bolstering organizational resilience and reputation. However, assessing the effectiveness of empathic communication is complex. Scholars like Jin and Ikeda (2023) propose various metrics, including verbal and nonverbal cues, active listening, and responsiveness. Yet, challenges arise in quantifying and comparing empathic behaviors across diverse demographics and organizational settings.

The importance of empathy extends beyond crisis communication; it’s integral to effective leadership. Indeed, Kock et al. (2019) define empathetic leadership as leadership that “focuses on the emotional relationship between a leader and follower—how much a leader understands a follower’s work situation, invests in emotional understanding, and provides emotional security for the follower” (p. 217), the definition we follow in this work. Various theories highlight empathy as a vital trait for leaders, enabling them to consider individual needs, recognize emotions in others, and navigate complex contexts. Furthermore, empathetic leaders tend to connect better with stakeholders and are perceived as more effective performers (Dolamore, 2021; Sadri et al., 2011).

Servant leadership refers to a values-oriented approach where leaders focus primarily on serving followers’ needs and development, using ethical influence to enhance individual growth, collective well-being, and long-term organizational effectiveness (Greenleaf, 1977). This is a model that prioritizes empathy, is characterized by traits like listening, healing, awareness, and commitment to growth (Spears, 2002). Central to this is empathy, where leaders strive to understand others’ perspectives, validating their experiences and fostering a sense of uniqueness. Building on this, researchers identify dimensions of servant leadership, like emotional healing, closely tied to empathy (Liden et al., 2014). Emotional healing involves sensitivity to others’ concerns and a willingness to address them, as validated by scales developed by Liden et al. (2014). In this context, successful empathetic leadership should base its behavior on principles (like honesty, openness, and responsibility) that serve as a model and offer stability during challenging situations (Deliu, 2019).

Xie and Johns (2020) further support the pivotal role of empathetic leadership in crisis contexts, emphasizing its contribution to trust-building and uncertainty reduction. Similarly, J. Wang and Chrobot-Mason (2019) stress how empathy enables leaders to meet the emotional needs of employees during challenging times. Yet, perceived empathy varies across leaders and contexts. Geographical and cultural factors influence its expression and reception, as evidenced by studies on Eastern and Western cultures (Smith et al., 2019; Muss et al., 2026). Moreover, organizational excellence correlates with higher levels of perceived empathy among communication leaders, underscoring its strategic importance (Muss et al., 2026).

Building on these insights, it becomes essential to consider how empathetic leadership is embedded within organizational culture, as culture not only frames leaders’ behaviors but also conditions how employees interpret and respond to them (Kwantes & Boglarsky, 2007).

### 2.2. Organizational Culture

Empathetic communication styles vary depending on the type of organization. The organization’s nature, industry, culture, and stakeholders all play a role in shaping communication strategies, particularly during times of crisis. For example, a study by Park et al. (2018) highlights that nonprofit organizations prioritize empathy and emotional connection in their communication efforts to cultivate donor support and community engagement. Conversely, corporations may opt for a more strategic approach to empathy, balancing organizational objectives with stakeholder needs (Grunig & Repper, 2013). This underscores the importance of tailoring communication strategies to reflect organizational values and goals. Importantly, while organizational culture is often treated as primarily relevant to internal communication, public relations scholarship demonstrates that external communication is deeply rooted in internal cultural values, norms, and assumptions, as organizations project their internal culture outward through stakeholder communication and relationship management practices (Grunig, 2013).

Moreover, organizational culture influences how leaders communicate. Worline and Dutton (2017) suggest that organizations emphasizing empathy and support tend to foster empathetic leadership behaviors. In contrast, organizations with hierarchical structures may struggle to promote empathetic communication due to power dynamics (Cameron & Caza, 2004).

Perceived empathy among communication leaders also varies based on the type of organization and hierarchical level. Morehouse (2007) found that leaders in nonprofit sectors exhibited higher overall levels of emotional intelligence, such as empathy, compared to their counterparts in for-profit sectors. Additionally, hierarchical level within organizations affects the display of empathy, with senior executives often experiencing greater pressure and scrutiny that can impact their ability to communicate empathetically (Smith et al., 2019). Thus, understanding these factors is crucial for effectively crafting empathetic communication strategies that align with organizational values and meet stakeholder needs.

### 2.3. Excellent Organizations

Empathy holds a pivotal role in effective leadership within the realm of communication. According to Jandt (2017), empathetic communication necessitates both cognitive understanding and emotional responsiveness, enabling leaders to establish profound connections with their team members. By actively listening, acknowledging emotions, and demonstrating comprehension, empathetic leaders cultivate a supportive atmosphere conducive to collaboration and innovation (Dutton & Heaphy, 2003). Additionally, empathetic communication fosters psychological safety, encouraging diverse perspectives and contributions to strategic discussions (Edmondson, 1999).

In the domain of organizational strategic planning, empathetic leadership proves particularly invaluable. Research by Cameron and Caza (2004) indicates that empathetic leaders foster a sense of belonging and inclusivity, enhancing team cohesion and commitment to shared objectives. During high-level meetings, these leaders facilitate open dialogue and constructive feedback, leveraging diverse viewpoints to inform decision-making processes (Gardner, 2011). Furthermore, adept conflict management and tension resolution by empathetic leaders mitigate resistance to change and promote organizational agility (Barsade, 2002).

Numerous empirical studies underscore the positive ramifications of empathetic leadership on organizational outcomes. For instance, Men et al. (2021) delve into organizations renowned for their effective communication practices, identifying empathy as a cornerstone in establishing a supportive and transparent communication culture. Organizational excellence encompasses transparency, authenticity, and stakeholder engagement, all facilitated by empathetic communication (Grunig, 2013). Supporting this notion, Mehra and Srivastava (2024) demonstrate a positive correlation between organizational communication effectiveness and empathetic leadership behaviors. Organizations prioritizing open, empathetic communication tend to enjoy heightened levels of employee satisfaction, trust, and loyalty (Grunig & Repper, 2013), thereby bolstering their reputation and competitiveness in the market.

This interplay between organizational type, culture, and leadership empathy highlights the broader question of how empathetic leadership contributes to organizational excellence, a theme central to communication scholarship (Men & Stacks, 2013). Building on the understanding of how empathetic leadership fosters organizational effectiveness, it is equally important to consider how gender shapes both the expression and perception of empathy, influencing leadership dynamics and employee experiences within communication settings (Eagly & Carli, 2003).

### 2.4. Gender Differences in Empathetic Leadership

Various studies have delved into the role of gender in shaping empathetic leadership, examining competencies, styles, perception, and expression. Y. Wang and Liu (2022) stress the developmental aspect of empathetic leadership skills concerning gender, arguing that societal expectations and socialization processes often lead women to display higher levels of empathy. Research on gender and leadership stereotypes suggests that women are often associated with more relational, person-oriented leadership, whereas men are linked to more task-oriented, agentic styles (Tremmel & Wahl, 2023). Additionally, gender stereotypes impact how empathetic leadership is perceived, with women often facing higher expectations and scrutiny, (Vongas & Al Hajj, 2015).

Additionally, García-Rico et al. (2021) highlight contextual factors like organizational culture in shaping gender-specific leadership behaviors, advocating for environments that value empathetic leadership to mitigate gender disparities. A meta-analysis conducted by Lee et al. (2020) revealed that women tend to possess higher emotional intelligence, including empathy, which can affect their effectiveness in empathetic leadership, a trait often observed in female communication leaders, attributed to societal norms (Eagly et al., 2003).

However, Hojat et al. (2002) suggest that the relationship between gender and empathy in leadership communication is more complex than commonly assumed. Building on this, Eagly and Carli (2003) note that women often demonstrate a stronger tendency toward empathetic communication styles. Similarly, Sanchez-Burks et al. (2018) emphasize that female leaders are frequently perceived as more empathetic and approachable. Taken together, these findings point to persistent gender disparities in empathy perceptions, with female leaders consistently viewed as more empathetic (L. Wang et al., 2021). However, societal norms and stereotypes may distort these perceptions, emphasizing the necessity for a nuanced examination of the interplay between gender and empathy in communication. Understanding how gender influences the expression and perception of empathy provides a foundation for examining the downstream effects of empathetic leadership on employee outcomes, particularly engagement, commitment, and motivation in organizational contexts (Breevaart et al., 2014).

### 2.5. Employee Engagement

In this section of literature review, we focus on a few key behavioral outcomes as related to the practice of empathetic leaderhship in corporate communications.

Employee engagement, as defined by Kahn (1990), refers to the emotional commitment, dedication, and involvement employees have towards their work and organization. Recognizing its importance, organizations are increasingly focusing on fostering employee engagement as a strategic priority to improve productivity, innovation, and overall success.

Leadership empathy plays a significant role in influencing employee engagement and organizational outcomes. Research by Miao et al. (2019) suggests that empathetic leaders positively impact employee engagement levels through trust and perceived organizational support. Similarly, Meyer and Allen (2018) found that empathetic communication from leaders enhances employees’ emotional attachment to the organization and encourages them to go beyond their job requirements. Furthermore, studies by Liden et al. (2014) highlight the positive correlation between leader empathy and follower engagement, particularly through relational identification and job meaningfulness.

Effective communication infused with empathy is crucial for enhancing employee engagement, according to Day et al. (2014). They emphasize the role of empathetic communication in creating a sense of belonging and intrinsic motivation among employees. Galinsky et al. (2008) also found that empathetic leaders establish supportive and inclusive workplace environments, leading to increased job satisfaction, organizational commitment, and performance, as supported by research by Eisenbeiss et al. (2008).

Research by Mehra and Srivastava (2024), further underscores the importance of empathy in communication for strengthening connections between leaders and employees. Empathetic communication fosters a supportive environment where employees feel valued, heard, and motivated, ultimately enhancing their commitment to organizational goals.

Moreover, empathy in communication positively correlates with employees’ affective organizational commitment, especially in flexible or remote work environments. Studies by Y. Wang and Lee (2018) and Kim et al. (2022) demonstrate that leaders who demonstrate empathy cultivate a sense of belonging and loyalty among employees, reinforcing their emotional connection to the organization and commitment to its values and objectives. Empathetic communication builds trust, transparency, and organizational commitment among employees, contributing to overall organizational success. Building on the positive effects of empathetic leadership on engagement, recent studies underscore the pivotal role of empathetic leadership in mitigating burnout, thereby enhancing employee engagement and fostering a resilient organizational culture (Muss et al., 2026).

### 2.6. Burnout Reduction

Empathy plays a pivotal role in enhancing workplace communication, particularly within dynamic industries where creativity, collaboration, and emotional intelligence are paramount. For example, Kock et al. (2019) found that empathetic leaders—those who listen, understand, and provide supportive communication—enhance follower job satisfaction and performance. More recent work by Ma et al. (2024) further shows that empathetic leadership strengthens psychological safety, which in turn promotes innovative behaviour and more open, collaborative communication within teams. This sentiment is echoed in studies by Smith et al. (2019) and Jones and Lee (2020), which reveal that employees who perceive empathy from their supervisors and colleagues are less likely to experience burnout.

Furthermore, empathetic communication fosters a supportive work environment, serving as a buffer against stressors that could otherwise impact employee well-being (L. Wang & Chang, 2018). Conversely, communication lacking in empathy can contribute to burnout, as emphasized by Maslach and Leiter (2017) and corroborated by Lee et al. (2020) and Rodriguez and Gonzalez (2019). Leaders who neglect empathy risk exacerbating workplace tensions and worsening burnout rates, underlining the importance of empathetic communication in promoting employee welfare.

Numerous studies highlight the positive impact of empathetic leadership on reducing burnout among employees (Smith et al., 2019; Muss et al., 2026). In the communication industry, characterized by tight deadlines and high client expectations, burnout is prevalent (Gascón et al., 2021). Empathetic leadership has been shown to play a meaningful role in shaping positive employee experiences and organizational outcomes. Kock et al. (2019) found that leaders who demonstrate emotional support and understanding foster stronger relational connections with employees, which in turn enhances follower performance and overall workplace functioning. Their study highlights that empathetic behaviors, such as attentive listening, validating employee concerns, and responding with genuine care, contribute to a more supportive organizational climate. These findings underscore the importance of empathy as a core leadership competency in reducing stress and promoting healthier interactions within teams. Building on the evidence that empathetic leadership reduces employee burnout, research also indicates that such leadership can significantly decrease turnover intentions, as employees who perceive support and understanding from their leaders are more likely to remain committed to their organizations (Trinkenreich et al., 2023; Daniels, 2025).

### 2.7. Turnover Intention

Turnover rates in the communications industry are frequently linked to factors such as job stress, burnout, and dissatisfaction with work conditions (Müller et al., 2012). Moreover, turnover intention, a key measure of how likely employees are to leave their current job, serves as a crucial indicator of employee retention. (Hom et al., 2012). Notably, turnover intention not only affects organizational performance but also imposes considerable costs related to recruitment and training (Mobley et al., 1979).

Research underscores that empathetic communication within leadership can mitigate employees’ turnover intentions from the perspective of effective internal communicaiton. For example, Yue et al. (2023) found that empathetic leadership communication significantly mitigates employees’ turnover intention by creating a supportive environment in which employees feel understood and valued. Furthermore, empirical studies in leadership research have highlighted the positive association between empathetic leadership and decreased turnover intention among employees. For instance, Wibowo and Paramita (2022) investigated nurses whose specific duties were to provide care to patients during COVID-19. Their research confirmed that the applications of mindful and empathetic leadership helped reduce turnover intention and increase resilience. Dutton and Heaphy (2003) found that employees perceiving their leaders as empathetic were less inclined to consider leaving their positions. Similarly, a meta-analysis by Miao et al. (2019) demonstrated a substantial negative relationship between empathetic leadership and turnover intention across various sectors, including communications. Such empirical research confirmed the important relationship between empathetic leadership and turnover intention and employee resilience, which inspires our application of such prediction to a communication setting in this study.

Moreover, empathetic leaders not only offer emotional support and cultivate a sense of belonging but also alleviate job-related stressors, strengthening organizational commitment (Dutton et al., 2006; Lilius et al., 2011). Consequently, employees tend to remain loyal to their organization and are less likely to explore alternative job opportunities. Research shows that empathetic leadership correlates with increased employee engagement and job satisfaction, crucial factors in decreasing turnover intention (Eisenberger et al., 1986).

Given the importance of empathetic communication in leadership within the PR industry and its implications for organizational outcomes such as employee engagement, burnout, and turnover intention, it is essential to examine how empathetic leadership varies across different contexts and its impact on these critical factors. Understanding the nuanced interplay between perceived empathy and leadership effectiveness, along with the role of gender, organizational type, and hierarchical level, will offer valuable insights into how empathetic leadership can enhance organizational success. Therefore, we propose the following research questions and hypotheses to guide our research:

**RQ1.**  *How do communication professionals perceive senior leaders’ empathy, and how does it vary by gender, country, organizational type, excellence, and hierarchical level?*

**RQ2.**  *To what extent do communication professionals perceive an increase in empathic communication from leaders over the past year, and how does it vary by gender, hierarchical level, age, experience, and country?*

**RQ3.**  *How effective do communication professionals perceive senior leaders’ demonstration of empathy, and how does it vary by gender, hierarchical level, age, experience, and country?*

**H1.**  *Female communication leaders are perceived as demonstrating higher levels of empathy compared to their male counterparts.*

**H2.**  *The practice of empathetic leadership is positively associated with employee engagement.*

**H3.**  *The practice of empathetic leadership is negatively associated with employee burnout.*

**H4.**  *The practice of empathetic leadership is negatively associated with employees’ intentions to leave the organization (turnover intentions).*

## 3. Research Method

### 3.1. Online Survey and Sampling

An international online survey was performed as part of a two-year-long global research initiative evaluating leadership and organizational dynamics within the communication and public relations sector, specifically targeting full-time communication professionals working in public relations, advertising, communication management, and other related areas in the broad communicaion profession in Canada and the United States. We used Qulatrics online survey platform to program our online survey. An audience-panel approach was used to ensure the quality of sample, as well as to recruit the targeted full-time communication professionals in targeted markets. Prior to beginning data collection, the study was reviewed by the Institutional Review Board (IRB) at the principal investigator’s university and determined to be exempt, given the fact that this is continued research from the previous edition of the global communication research project. The actual data collection of this online survey took place over a period of three months, starting in mid-August and concluding at the end of November 2023.

The data collection and recruitment of participants were managed by Qualtrics once the survey was launched. Based on the recorded information, the survey link received a total of 7272 clicks by potential participants. However, we designed a series of questions at the beginning of the online survey to function as the filter questions to screen out unqualified participants. A sample filter question is “what is your current employement status.” When participants selected the answer key of “employed full-time,” they were advanced to the next screening question. Participants who passed all screening questions were retained to advance to the main questionnaire of the survey.

We deliberately designed those screening questions to ensure the quality of our sample. After the screening questions and data cleaning, we were able to record a total of 1055 valid and complete responses for final data analysis. Most survey items were measured using standardized Likert-type scales (i.e., 1–5 or 1–7, depending on the questions), allowing respondents to indicate their level of agreement or frequency across key constructs related to leadership, employee experiences, and organizational outcomes. Demographic questions were also included at the end of the questionnaire to help us achieve the sample quotas.

### 3.2. Measures of Tested Variables

#### 3.2.1. Excellence Orientation

This reflects the extent to which communication departments meet professional standards associated with strategic influence and performance. This construct was operationalized using four dimensions assessing (1) advisory influence, (2) executive influence, (3) perceived success, and (4) professional competence, consistent with excellence theory in public relations, adapted from Tench and associates’ work on building excellent communication departments (Tench et al., 2017). A sample item is “in your organization, how seriously do senior leaders take the recommendations of the communication function?” The answer keys ranged form 1 (not seriously at all) to 7 (very seriously).

Respondents evaluated their department’s performance on each dimension using a 7-point Likert scale. Item responses were averaged to create a composite index of excellence orientation, with higher scores indicating stronger perceptions of excellence in communication. Departments were classified as meeting the criteria for excellence if they scored six or higher across all four dimensions, following prior operational conventions in excellence communication research (Tench et al., 2017). Based on this criterion, 35.1% of departments met the standard for excellence. The scale demonstrated good internal consistency (Cronbach’s α = 0.82).

#### 3.2.2. Empathetic Communication

Perceived leader empathy was operationalized using two items capturing employees’ overall assessment of empathetic communication behaviors displayed by their immediate leaders. Respondents were asked to indicate the extent to which they agreed that their leader is empathic when communicating with employees and that their leader has increased the level of empathy in their communication over the past year. Responses were recorded on a 5-point Likert scale ranging from 1 (strongly disagree) to 5 (strongly agree). Item responses were averaged to create a composite index of perceived leader empathy, with higher scores indicating stronger perceptions of empathetic leadership. The two-item scale demonstrated acceptable internal consistency (Cronbach’s α = 0.73).

Items were conceptually informed by prior leadership and empathy literature and adapted to the communication and PR context in this study. Specifically, we adapted the two measurement items based on the servant leadership research of Greenleaf (1970) and Liden and associates (Liden et al., 2015). Descriptively, 71.2% of communication professionals agreed that their leaders demonstrate empathy in their communication with employees, while 67.9% reported that their leaders have increased the level of empathy in their communication over the past year.

#### 3.2.3. Leadership Empathy

To further examine empathy as enacted behavior, a separate index focused on observable empathetic leadership practices was adapted from Boyatzis et al.’s (2000) Emotional Competence Inventory. Respondents indicated whether their leaders regularly engaged in specific empathetic behaviors, including: paying close attention and listening effectively, recognizing employees’ strengths and limitations, asking clarifying questions to ensure understanding, and demonstrating care for others’ personal well-being. Responses were recorded on a 5-point Likert scale ranging from 1 (strongly disagree) to 5 (strongly agree). These items were aggregated into a behavioral empathy index, which demonstrated strong internal consistency (Cronbach’s α = 0.82). The most frequently reported behavior was active listening (74.9%).

#### 3.2.4. Employee Engagement

Employee engagement reflects employees’ emotional and cognitive investment in their work and organization as outlined in Schaufeli and Bakker’s (2004) research on work engagement, which is one of the most frequently cited works regarding the job demands-resources model. This construct was measured using items assessing: organizational belonging and attachment, sense of purpose. enthusiasm for work, work engagement, and energy and vitality.

Items were adapted from Schaufeli and Bakker’s (2004) research, which is commonly used in organizational communication research. The engagement scale showed marginal but acceptable reliability (Cronbach’s α = 0.67), consistent with prior research using short-form engagement indices.

#### 3.2.5. Mental Health

Mental health was measured using items informed by the well-being dimensions proposed by Van Dierendonck et al. (2001), focusing on emotional exhaustion and perceived coping capacity. Nearly half of respondents (46.1%) reported feeling emotionally drained at the end of the workday, while 76.9% indicated confidence in their ability to manage work-related challenges. The measures demonstrated acceptable internal consistency (Cronbach’s α = 0.72).

#### 3.2.6. Turnover Intention

Turnover intention captures employees’ conscious and deliberate willingness to leave their current organization within a specified time frame (Van Veldhoven & Meijman, 1994). This construct was measured using items assessing respondents’ likelihood of seeking alternative employment within the next year, a widely used indicator of organizational instability and employee dissatisfaction in organizational research (Van Veldhoven & Meijman, 1994).

Respondents indicated their agreement with turnover-related statements on a 5-point Likert scale (1 = strongly disagree, 5 = strongly agree), with higher scores reflecting stronger intentions to leave their current organization. Results indicate that more than one-third of communication professionals reported intentions to change jobs within the next year, signaling a meaningful level of workforce volatility within the communication and public relations sector. The turnover intention scale demonstrated satisfactory reliability (Cronbach’s α = 0.70).

#### 3.2.7. Sample Comparison and Data Aggregation

Independent-samples *t*-tests were conducted to assess differences between U.S. and Canadian respondents across all major study variables. No statistically significant differences were observed. Consequently, the samples were merged to increase statistical power and improve the stability of subsequent analyses. All analyses were conducted using the combined sample of 1055 full-time communication professionals.

## 4. Results

### 4.1. Participants’ Demographics

The final data set used for analysis in this study comprised 797 full-time communication professionals from the United States and 258 from Canada. In the U.S. sample, there were 392 women (49.3%) and 400 men (50.2%); while in the Canadian sample, there were 126 women (48.8%) and 132 men (51.2%). Core demographic variables including years of professional experience, organizarional hierarchical level within the organization, types of organization, age group, and level of organizational excellence.

Concerning professional experience, 48.9% of respondents had over a decade of experience, with 26.8% having 6 to 10 years, and 24.3% having less than 5 years of experience. In terms of organizational hierarchy, 50.5% of respondents held middle-level hierarchical roles, such as team or unit leaders. This was followed by 30.4% occupying top-level roles within their organizations, while 19% were in lower hierarchical level as team members. In terms of industry sectors, the majority of respondents worked in the private sector (44%), followed by publicly held companies (19.5%), government/public sector or political organizations (15.4%), communication/public relations agencies (14.1%), and non-profit organizations (7.0%).

In terms of age distribution, the largest group was between 30 and 39 years old (38.6%), followed by those aged 18 to 29 (24.7%), 40 to 49 (22.8%), and those over 50 (13.7%). Communication departments were selected based on their organizational excellence scores, and statistical methods were used to distinguish between excellent and non-excellent departments.

### 4.2. RQ1: Perceived Empathy from Senior Communicators

The primary objective of our initial research question was to assess the variability in empathy perception toward senior communication leaders. Within the research design framework, we surveyed communication professionals regarding their communication leaders’ level of empathy. As Table 1 shows, several notable differences in perceptions emerged across the key demographic variables of gender, country, organizational excellence, type of organization, and hierarchical position. Specifically, male professionals reported significantly higher levels of agreement regarding the empathy exhibited by their leaders compared to their female counterparts, *F*(1, 1049) = 16.55, *t* = −3.65, *p* < 0.001, (*M* = 3.95, *SD* = 0.83) versus (*M* = 3.76, *SD* = 0.93), respectively. As for country differences, no significant difference was found between U.S.-based (*M* = 3.86, *SD* = 0.91) and Canada-based (*M* = 3.85, *SD* = 0.83) respondents, *t*(1053) = 0.18, *p* = 0.861.

Organizations that achieved excellence, defined as scoring six or higher across all four dimensions, as outlined in the methods, also showed a significant difference in perceived empathy, *F*(1, 904) = 26.11, *p* < 0.001, (*M* = 4.29, *SD* = 0.69). Additionally, significant differences in perceptions of empathy based on type of organization, *F*(5, 1049) = 4.40, *p* < 0.001. Respondents from private organizations (*M* = 3.98) reported the highest levels of agreement, followed by those from public (*M* = 3.84), agency (*M* = 3.80), nonprofit (*M* = 3.73), self-employed (*M* = 3.75), and government (*M* = 3.65) organizations. Post hoc LSD comparisons indicated significant differences between private and government (*p* < 0.001, Mean Diff. = 0.34), private and nonprofit (*p* = 0.022, Mean Diff. = 0.25), private and self-employed (*p* = 0.032, Mean Diff. = 0.24), and public and government (*p* = 0.039, Mean Diff. = 0.19) organizations.

Regarding hierarchical position, respondents at higher levels of the organization reported significantly greater perceptions of empathy, *F*(2, 1021) = 7.31, *p* < 0.001. Post hoc LSD tests revealed significant differences between low (*M* = 3.69) and high (*M* = 4.00) positions (*p* < 0.001, Mean Diff. = −0.31) and between mid (*M* = 3.84) and high (*M* = 4.00) positions (*p* = 0.009, Mean Diff. = 0.16), while the difference between low and mid levels was not statistically significant (*p* = 0.059).

### 4.3. RQ2: Perception If There Has Been an Increase in the Amount of Empathic Communication

Providing insights about the level of empathy perceived by communication professionals from senior communication leaders would fall short if we did not explore how empathic communication has evolved over time. Therefore, for our second research question, we examined perceptions of whether communication leaders had increased their empathic communication during the previous twelve months.

Table 2 indicates that male professionals (*M* = 4.00, *SD* = 0.67) reported significantly higher perceived levels of empathic communication from their leaders than female professionals (*M* = 3.77, *SD* = 0.80), *F*(1, 1049) = 19.06, *t* = −5.10, *p* < 0.001, Mean Diff. = −0.23.

Additionally, respondents at the highest hierarchical level (*M* = 4.07, *SD* = 0.71) expressed significantly stronger agreement compared to those at the lowest (*M* = 3.65, *SD* = 0.78) and middle (*M* = 3.86, *SD* = 0.71) levels, *F*(2, 1021) = 19.27, *p* < 0.001. Post hoc LSD tests showed significant differences between low and mid (*p* = 0.001, Mean Diff. = −0.21), low and high (*p* < 0.001, Mean Diff. = −0.42), and mid and high (*p* < 0.001, Mean Diff. = −0.21) positions.

When considering age groups, participants aged 18–29 years (*M* = 3.61, *SD* = 0.69) reported significantly lower agreement compared to those aged 30–39 years (*M* = 3.96, *SD* = 0.70), 40–49 years (*M* = 4.04, *SD* = 0.71), and 50 years and older (*M* = 3.95, *SD* = 0.87), *F*(3, 1051) = 17.42, *p* < 0.001. Post hoc LSD results confirmed all pairwise differences were significant (*p* < 0.001) with Mean Diffs. ranging from −0.33 to −0.42.

A similar pattern emerged for years of professional experience, *F*(3, 1051) = 13.40, *p* < 0.001. The most junior professionals—those with up to five years of experience (*M* = 3.66, *SD* = 0.71), reported significantly lower agreement that their communication leaders had improved in empathic communication compared to those with 6–10 years (*M* = 3.94, *SD* = 0.70; *p* < 0.001, Mean Diff. = −0.28), 11–15 years (*M* = 4.05, *SD* = 0.63; *p* < 0.001, Mean Diff. = −0.39), and 16 or more years (*M* = 3.90, *SD* = 0.91; *p* = 0.002, Mean Diff. = −0.24).

Finally, no significant difference was found between U.S.-based (*M* = 3.91, *SD* = 0.76) and Canada-based (*M* = 3.84, *SD* = 0.71) respondents, *t*(1053) = 1.33, *F*(1, 1053) = 1.19, *p* = 0.181, Mean Diff. = 0.07.

### 4.4. RQ3: The Impact of Empathy Measures Demonstrated by Top Communication Leaders

Our third research question aimed to evaluate the perception of communication professionals regarding how effectively their leaders have demonstrated empathy. Results presented in Table 3 reveal that male professionals (*M* = 4.00, *SD* = 0.72) reported significantly higher levels of perceived empathy communicated by their leaders, *F*(1, 1049) = 15.13, *t* = −4.73, *p* < 0.001, as compared to their female colleagues (*M* = 3.77, *SD* = 0.85). Additionally, respondents at the highest hierarchical level (*M* = 4.05, *SD* = 0.76) expressed significantly stronger agreement, *F*(2, 1021) = 15.05, *p* < 0.001, compared to those at the lowest (*M* = 3.66, *SD* = 0.82) and middle levels (*M* = 3.87, *SD* = 0.77). Post hoc LSD tests indicated significant differences between level 1 and 2 (*p* = 0.002, Mean Diff = −0.21), level 1 and 3 (*p* < 0.001, Mean Diff = −0.40), and level 2 and 3 (*p* < 0.001, Mean Diff = −0.19).

Similarly, when considering age groups, participants aged 18–29 years (*M* = 3.61, *SD* = 0.79) reported significantly lower perceptions of empathy, *F*(3, 1051) = 15.96, *p* < 0.001, than those aged 30–39 (*M* = 3.95, *SD* = 0.74), 40–49 (*M* = 4.05, *SD* = 0.74), and 50+ (*M* = 3.95, *SD* = 0.91). In terms of years of professional experience, significant differences also emerged, *F*(3, 1051) = 11.99, *p* < 0.001. Professionals with 16+ years of experience (*M* = 3.65, *SD* = 0.79) reported significantly lower perceptions of empathy compared to those with 6–10 years (*M* = 4.05, *SD* = 0.66), up to 5 years (*M* = 3.92, *SD* = 0.73), and 11–15 years (*M* = 3.90, *SD* = 0.95), as indicated by post hoc Tukey HSD comparisons (*p* < 0.001, Mean Diff. = −0.26; *p* < 0.001, Mean Diff. = −0.40; *p* = 0.003, Mean Diff. = −0.25, respectively).

Lastly, no significant difference was found between U.S.-based (*M* = 3.90, *SD* = 0.81) and Canada-based (*M* = 3.86, *SD*= 0.76) respondents, *F*(1, 1053) = 2.33, *t* = 0.78, *p* = 0.435.

### 4.5. The Application of Empathetc Leadership Across Different Variables

In this section, we aimed to evaluate whether empathetic leadership was positively, negatively, or not significantly associated with employee engagement, employee burnout, and turnover intentions (see Table 4).

For **H1**, the analysis revealed a statistically significant relationship between leader gender and perceived leader empathy. However, contrary to the hypothesis, the findings indicate that male leaders (*M* = 3.99, *SD* = 0.72) are perceived as demonstrating greater empathy than female leaders (*M* = 3.76, *SD* = 0.87). This difference is supported by the statistical analysis (*F*(1, 1007) = 21.34, *p* < 0.001). These results suggest that perceived empathy in leadership communication was rated higher for male leaders than for female leaders.

For **H2**, the analysis revealed a significant positive correlation between empathetic leadership and employee engagement (*r* = 0.591, *p* < 0.001). This indicates that higher levels of empathy displayed by leaders are associated with greater employee engagement.

For **H3**, the results indicated no significant relationship between empathetic leadership and mental health (*r* = 0.057, *p* = 0.065). This suggests that empathetic leadership did not significantly influence employees’ mental health, either positively or negatively. Further research may be needed to identify other potential factors that shape this relationship.

Lastly, for **H4**, we examined the correlation between empathetic leadership and turnover intention. The analysis revealed a significant positive association (*r* = 0.662, *p* < 0.001). This indicates that higher levels of empathetic leadership are strongly related to lower turnover intention, suggesting that empathy may foster employees’ commitment and retention.

## 5. Discussion

The findings of this study provide critical insights into how communication professionals perceive the empathy of their senior communication leaders and the broader organizational implications of these perceptions. By synthesizing the outcomes of the research questions, this discussion offers a nuanced understanding of the role empathy plays in professional communication settings, highlighting both its strengths and its limitations as a leadership practice.

### 5.1. Perceived Empathy from Senior Communication Leaders (RQ1)

The first research question examined how communication professionals perceive the empathy of their senior leaders. Overall, respondents reported a generally positive perception of empathic leadership, with no statistically significant differences across geographical lines between the United States and Canada. This pattern is consistent with the view that empathy, as a leadership competency, transcends regional cultural contexts within North America and is broadly recognized as a desirable trait in leadership communication (Shuck & Reio, 2014; Avolio & Bass, 1995).

However, the emergence of gender disparities complicates this otherwise positive pattern of results. In our data, male professionals reported significantly higher levels of perceived empathy than female professionals, suggesting that empathy may not be equally experienced across genders. This resonates with existing servant leadership research (Greenleaf, 1970; Liden et al., 2015), which shows that women often expect greater relational sensitivity in leadership and may be more attuned to subtle deficits in empathic behavior (Eagly & Carli, 2007; Fischer, 2000). Men, conversely, may interpret general supportive gestures as empathic without requiring the depth of relational engagement expected by women (Ciarrochi et al., 2000). These dynamics suggest that gender may not merely be a demographic variable but an interpretive lens that shapes the evaluation of leadership behaviors.

As confirmed in our results, organizational type and hierarchical level were also significantly correlated with perceptions of empathy. Employees in the private sector reported the highest levels of empathy from their leaders, potentially reflecting organizational cultures that link relational leadership with performance, innovation, and competitive advantage. In contrast, public and nonprofit organizations may experience structural or resource constraints that limit leaders’ capacity to demonstrate empathy consistently (Boyatzis, 2018). Additionally, respondents at higher hierarchical levels reported greater empathy than those at lower levels, which may reflect both the selective demonstration of empathy toward senior colleagues and the interpretive privilege of higher-ranking professionals who interact more closely with leaders (Kahn, 1990; Aggarwal, 2022). These findings reinforce the notion that empathy is not evenly distributed across organizational strata and may be contingent upon access to leadership networks.

### 5.2. Perception of Increased Empathic Communication (RQ2)

The second research question explored whether communication professionals reported an increase in empathic communication from their leaders over the past year. Male professionals, older employees, and those at higher hierarchical levels reported the greatest perceived increases. This finding suggests that professional maturity and organizational tenure may enhance the ability to recognize, interpret, or value empathic communication (Ashkanasy & Daus, 2005). Younger and less experienced employees, in contrast, may either lack the interpretive frameworks to identify empathic cues or may enter the profession with heightened expectations for empathy that are not consistently met.

The absence of regional differences between U.S. and Canadian respondents further reinforces that these perceptions are shaped primarily by professional and organizational norms rather than national cultural contexts. This aligns with broader leadership research suggesting that empathy is a universally valued leadership trait but one that manifests differently depending on workplace demographics and role expectations (Goleman, 1995; Côté, 2014).

### 5.3. Impact of Empathy Demonstrated by Senior Communication Leaders (RQ3)

The third research question was focused on the perceived effectiveness of leaders’ empathic behaviors. Consistent with earlier findings, male professionals and those at higher organizational levels rated empathy as more effective, with age and tenure again emerging as moderating factors. This suggests that demographic variables may shape not only the recognition of empathy but also its perceived value.

These results underscore the importance of adaptive leadership—leaders cannot assume that empathy will be interpreted uniformly across employee groups. Instead, they must calibrate their empathic communication styles to accommodate diverse expectations and needs (Heifetz et al., 2009). For communication professionals working in a fast-paced and high-pressure industry, where workloads are heavy and crises frequent (Berger, 2019), empathic leadership may be particularly salient. However, the high-stakes, time-sensitive nature of the field also imposes structural constraints on how consistently leaders can demonstrate relational sensitivity. This tension underscores both the potential and the limits of empathy in the communication industry.

### 5.4. Application of Empathy with Organizational Outcomes (Hs)

The final research question explored the organizational consequences of perceived empathy. The study identified a strong positive association between empathy and employee engagement, aligning with prior research that connects empathic leadership to increased motivation, trust, and commitment (Kahn, 1990; Shuck & Reio, 2014). This finding further suggests that empathy remains a critical lever for enhancing employee involvement and cultivating organizational climates characterized by psychological safety and collaboration.

Yet, the absence of a significant relationship between empathy and burnout complicates this narrative. The communication industry is marked by intense work demands, client pressures, and the need for constant availability, all of which are structural contributors to burnout (Maslach & Leiter, 2017). While empathy can create a more supportive climate, it is unlikely to offset systemic stressors without complementary organizational strategies such as workload management, structural flexibility, or wellness interventions. This suggests that empathy, though valuable, should not be treated as a substitute for addressing organizational design and employee health.

Perhaps the most paradoxical finding is the positive association between perceived empathy and turnover intentions. This suggests that while employees may value empathy, it does not override dissatisfaction with other organizational factors such as career advancement, compensation, and fairness (Hom et al., 2017). In fact, empathy may heighten employees’ awareness of discrepancies between what leaders express and what the organization delivers. For instance, leaders may convey understanding of employee struggles without being able to implement structural changes, inadvertently reinforcing frustration. This highlights that empathy may work best when it is embedded within a broader organizational strategy that integrates structural support, equitable policies, and clear career pathways if it is to contribute to long-term retention.

### 5.5. Integrative Implications

Taken together, these findings underscore the complexity of empathy as both a leadership practice and an organizational outcome. While empathy was positively associated with engagement, its uneven reception across gender, age, and hierarchical levels suggests it cannot be assumed to function uniformly within diverse workforces. Moreover, its limited association with mitigating burnout and its paradoxical link to turnover intentions highlight a possible limitations in high-pressure environments such as the communication industry. We would argue that empathy is necessary but insufficient on its own—it must be accompanied by structural interventions, organizational reform, and transparent career development strategies.

This study contributes to the growing literature on empathetic leadership by situating its findings within the unique context of the communication industry. Unlike generic leadership studies, this research captures the nuances of a sector where speed, workload, and external pressures shape both leadership practices and employee expectations. In doing so, it extends prior research by suggesting that empathy’s effects are contingent on both demographic factors and industry-specific dynamics. Future research should therefore adopt intersectional and industry-sensitive approaches that examine how empathy interacts with systemic inequalities, professional pressures, and organizational design to shape employee well-being and performance.

### 5.6. Theoretical Implications

With the above pattern of results in mind, this study contributes to leadership and communication theory in three keyways. First, it reinforces the centrality of empathy as a leadership competency, while calling for frameworks that account for demographic variation in how empathy is perceived and valued. Leadership theories that fail to consider gendered and hierarchical dynamics risk overgeneralizing the effectiveness of empathy in practice.

Second, the study complicates the link between empathy and employee well-being. While prior research has positioned empathy as a buffer against disengagement and dissatisfaction, this study suggests that empathy alone cannot address systemic drivers of burnout or turnover (Maslach & Leiter, 2017; Hom et al., 2017). Future theoretical models should therefore integrate empathy into more comprehensive accounts of employee well-being that consider both relational and structural dimensions.

Third, the findings highlight empathy as a multidimensional construct influenced by organizational context. Sectoral and hierarchical differences point to the importance of embedding empathy within leadership models that are adaptable to diverse institutional logics and organizational cultures (Boyatzis et al., 2000; Aggarwal, 2022).

### 5.7. Practical Implications

The study also carries significant implications for practice, particularly within PR organizations where relational communication and trust are central to professional success. First, it underscores the need for leadership development programs that cultivate empathy as a core communicative skill. Such programs should be intentionally designed to address gendered expectations and cross-hierarchical communication, ensuring that empathy is effectively demonstrated to all employees, not only to peers or senior colleagues.

Second, the findings suggest that PR leaders should adopt tailored communication approaches that reflect the diverse needs of their employees. By actively seeking feedback from different demographic groups, leaders can mitigate disparities in how empathy is experienced and foster more inclusive organizational climates.

Third, empathy should be incorporated into broader well-being strategies that extend beyond relational support. Initiatives such as flexible work arrangements, access to mental health resources, mentorship opportunities, and transparent career pathways can complement empathic leadership and address the deeper organizational drivers of burnout and turnover.

Finally, the study points to the potential value of cross-sector learning. The higher levels of perceived empathy in the private sector suggest that public and nonprofit organizations may benefit from adapting private-sector practices that prioritize empathetic communication, while still tailoring them to their unique missions and constraints.

## 6. Conclusions

Overall, this study advances the understanding of empathy as a central dimension of leadership in organizational communication, with particular relevance for the public relations (PR) and communication industries. The findings underscore empathy’s potential to enhance employee engagement and foster relational trust, while simultaneously revealing its limitations as a singular solution to complex organizational challenges such as burnout and turnover. By highlighting how demographic factors—including gender, age, hierarchical position, and sector—shape the perception and effectiveness of empathic leadership, this study contributes to a more nuanced and contextually sensitive account of empathy in organizational life.

The results affirm that empathy is not experienced uniformly across professional groups. Male professionals, senior-level employees, and those in the private sector consistently reported more positive perceptions of empathic leadership, suggesting that empathy is filtered through existing workplace structures and power dynamics. These insights point to the necessity of interrogating empathy not merely as a universal trait of effective leadership but as a socially constructed and context-dependent phenomenon (Fletcher, 2004; Eagly & Carli, 2007). Leaders must therefore recognize that the reception of empathy is contingent on the social positions, expectations, and lived experiences of employees.

Importantly, the study also demonstrated that while empathy strongly predicts engagement, it does not independently buffer against burnout or prevent turnover. These findings highlight the complexity of organizational well-being, where empathic leadership is most effective when embedded within broader systemic strategies that include mental health resources, structural flexibility, and equitable career development opportunities. Without such complementary measures, empathy may risk being perceived as symbolic or insufficient, particularly by employees whose needs extend beyond relational communication.

### Limitations and Future Research

In addition to the positive outcomes and contributions this study was able to identify and contribute to the broad literature of empathetic leadership and communication practices, we must admit there are several limitations. First, one of the major limitations is associated with sample and data collection. Since we had a cross-sectional data-collection approach, we can’t track and compare the different perceptions of empathetic leadership before and after the COVID-19. Since the data collection happened towards the end of 2023, there is a possibility that the application of empathetic leadership has become more common given the uncertainty and stress the pandemic has caused over the past few years. Therefore, we highly recommend future research to focus on longitudinal research that could diminish the potential value of that approach.

Secondly, it is unclear whether empathetic communication is the only factor leading to perception and behavior change among employees. Therefore, more comprehensive design in a complex organizational setting will be more helpful to investigate such complicated relationships.

As part of the limitations embedded in this current research, we highly recommend that future research should continue to refine the conceptualization of empathy in leadership, exploring its intersection with gender, organizational structure, and systemic inequalities, and identifying pathways for translating empathic leadership into enduring organizational resilience and employee well-being.

In sum, this study supports that empathy is both essential and insufficient as a leadership strategy. It enhances engagement and relational trust but must be understood as part of a holistic organizational framework that addresses structural, cultural, and demographic realities. For the PR and communication industries, where the interplay between leadership, trust, and relational communication is particularly salient, embedding empathy within comprehensive strategies of equity, well-being, and organizational design is critical for long-term sustainability.

## Figures and Tables

**Table 1:** Comparisons of Leadership Empathy Across Demographic and Organizational Variables.

Variable/Category	Test	*n*	*M*	*SD*	*F*	*t*	*df*	*p* (Two-Tailed)	Mean Diff.
**Gender**									
Female (1)	*t*-test	519	3.76	0.93	16.55	−3.65	1049	<0.001	−0.20
Male (2)		532	3.95	0.83					
**Country**									
United States	*t*-test	797	3.86	0.91	4.60	0.18	1053	0.861	0.01
Canada		258	3.85	0.83					
**Excellence Orientation**	*t*-test	318	4.29	0.69	26.11	−12.07	904	<0.001	−0.64
**Type of Organization**	ANOVA				4.40		5, 1049	<0.001	
Public		206	3.84	—					
Private		464	3.98	—					
Government		162	3.65	—					
Non-profit		74	3.73	—					
Agency		77	3.80	—					
Self-employed		72	3.75	—					
Post hoc (LSD) Public vs. Private								0.047	−0.15
Post hoc (LSD) Public vs. Government								0.039	0.19
Post hoc (LSD) Private vs. Government								<0.001	0.34
Post hoc (LSD) Private vs. Non-profit								0.022	0.25
Post hoc (LSD) Private vs. Self-employed								0.032	0.24
**Position Level**	ANOVA				7.31		2, 1021	<0.001	
1 (Low)		170	3.69	0.91					
2 (Mid)		533	3.84	0.86					
3 (High)		321	4.00	0.87					
Post hoc (LSD) 1 vs. 3								<0.001	−0.31
Post hoc (LSD) 1 vs. 2								0.059	−0.15
Post hoc (LSD) 2 vs. 3								0.009	0.16

Note. *M* = mean; *SD* = standard deviation. *p* values < 0.05 indicate statistical significance. All tests were two-tailed.

**Table 2:** Comparative Tests for Empathic Communication.

Variable/Category	Test	*n*	*M*	*SD*	*F*	*t*	*df*	*p* (Two-Tailed)	Mean Diff.
**Country**									
US	*t*-test	797	3.91	0.76	1.19	1.33	1053	0.181	0.071
Canada		258	3.84	0.71					
**Gender**									
Female (1)	*t*-test	519	3.77	0.80	19.06	−5.10	1049	<0.001	−0.23
Male (2)		532	4.00	0.67					
**Excellence Orientation**	*t*-test	318	4.31	0.61	8.25	−14.65	904	<0.001	−0.66
**Position Level**	ANOVA				19.27		2, 1021	<0.001	
1 (Low)		170	3.65	0.78					
2 (Mid)		533	3.86	0.71					
3 (High)		321	4.07	0.71					
Post hoc (LSD) 1 vs. 2								0.001	−0.21
Post hoc (LSD) 1 vs. 3								<0.001	−0.42
Post hoc (LSD) 2 vs. 3								<0.001	−0.21
**Age Group**	ANOVA				17.42		3, 1051	<0.001	
18–29 (1)		261	3.61	0.69					
30–39 (2)		408	3.96	0.70					
40–49 (3)		241	4.04	0.71					
50 + (4)		145	3.95	0.87					
Post hoc (LSD) 1 vs. 2								<0.001	−0.34
Post hoc (LSD) 1 vs. 3								<0.001	−0.42
Post hoc (LSD) 1 vs. 4								<0.001	−0.33
**Years of Experience**	ANOVA				13.40		3, 1051	<0.001	
Up to 5 years		256	3.66	0.71					
6–10 years		283	3.94	0.70					
11–15 years		289	4.05	0.63					
16+ years		227	3.90	0.91					
Post hoc (Tukey HSD) Up to 5 vs. 6–10								<0.001	−0.28
Post hoc (Tukey HSD) Up to 5 vs. 11–15								<0.001	−0.39
Post hoc (Tukey HSD) Up to 5 vs. 16+								0.002	−0.24

Note. *M* = mean; *SD* = standard deviation. All tests are two-tailed. *p* values < 0.05 are considered statistically significant.

**Table 3:** Independent Samples *t*-tests and ANOVA Results for Leadership Empathy.

Variable/Category	Test	*n*	*M*	*SD*	*F*	*t*	*df*	*p* (Two-Tailed)	Mean Diff.
**Country**									
US	*t*-test	797	3.90	0.81	2.33	0.78	1053	0.435	0.044
Canada		258	3.86	0.76					
**Gender**									
Female (1)	*t*-test	519	3.77	0.85	15.13	−4.73	1049	<0.001	−0.23
Male (2)		532	4.00	0.72					
**Excellence Orientation**	*t*-test	318	4.29	0.66	10.39	−12.89	904	<0.001	−0.63
**Position Level**	ANOVA				15.05		2, 1021	<0.001	
1 (Low)		170	3.66	0.82					
2 (Mid)		533	3.87	0.77					
3 (High)		321	4.05	0.76					
Post hoc (LSD) 1 vs. 2								0.002	−0.21
Post hoc (LSD) 1 vs. 3								<0.001	−0.40
Post hoc (LSD) 2 vs. 3								<0.001	−0.19
**Age Group**	ANOVA				15.96		3, 1051	<0.001	
18–29 (1)		261	3.61	0.79					
30–39 (2)		408	3.95	0.74					
40–49 (3)		241	4.05	0.74					
50+ (4)		145	3.95	0.91					
Post hoc (LSD) 1 vs. 2								<0.001	−0.34
Post hoc (LSD) 1 vs. 3								<0.001	−0.44
Post hoc (LSD) 1 vs. 4								<0.001	−0.35
**Years of Experience**	ANOVA				11.99		3, 1051	<0.001	
Up to 5 years		283	3.92	0.73					
6–10 years		289	4.05	0.66					
11–15 years		227	3.90	0.95					
16+ years		256	3.65	0.79					
Post hoc (Tukey HSD) 16> vs. others								<0.001	−0.26
Up to 5 vs. 6–10 years								<0.001	−0.40
Up to 5 vs. 11–15 years								0.003	−0.25

Note. *M* = mean; *SD* = standard deviation. *p* values < 0.05 indicate statistical significance. All tests are two-tailed.

**Table 4:** Summary of Statistical Tests for Hypotheses on Empathy and Organizational Variables.

Hs.	Variables/Comparison	Category	Test	*N*	*M*	*SD*	*F*	*df*	*p* (Two-Tailed)	Mean Diff.
**H1**	Female vs. Male Leaders (Empathy)	F	ANOVA	460	3.76	0.87	21.34	1–1007	<0.001	13.36
		M	ANOVA	540	3.99	0.72				
**H2**	Empathy and Employee Engagement	—	Pearson	1055	—	—	—	—	<0.001	—
									*r* = 0.591	
**H3**	Empathy and Mental Health	—	Pearson	1055	—	—	—	—	0.065	—
									*r* = 0.057	
**H4**	Empathy and Turnover Intention	—	Pearson	1055	—	—	—	—	<0.001	—
									*r* = 0.662	

Note. ANOVA = analysis of variance. Pearson = Pearson’s correlation coefficient. *p* < 0.05 indicates statistical significance.

## Data Availability

The raw data supporting the conclusions of this article will be made available by the authors on request.
